# Development of a Sustainable Flexible Humidity Sensor Based on *Tenebrio molitor* Larvae Biomass-Derived Chitosan

**DOI:** 10.3390/s25020575

**Published:** 2025-01-20

**Authors:** Ezekiel Edward Nettey-Oppong, Riaz Muhammad, Emmanuel Ackah, Hojun Yang, Ahmed Ali, Hyun-Woo Jeong, Seong-Wan Kim, Young-Seek Seok, Seung Ho Choi

**Affiliations:** 1 Department of Biomedical Engineering, Yonsei University, Wonju 26493, Republic of Korea; ezekieledward@yonsei.ac.kr (E.E.N.-O.); riaz@yonsei.ac.kr (R.M.); eackah.3@gmail.com (E.A.); yanghoo96@yonsei.ac.kr (H.Y.); alee@yonsei.ac.kr (A.A.); 2 Department of Electrical Engineering, Sukkur IBA University, Sukkur 65200, Pakistan; 3 Department of Biomedical Engineering, Eulji University, Seongnam 13135, Republic of Korea; hwjeong@eulji.ac.kr; 4 Department of Agricultural Biology, National Institute of Agricultural Sciences, Rural Development Administration, Wanju 55365, Republic of Korea; tarupa@korea.kr; 5 Gangwon-do Agricultural Product Registered Seed Station, Chuncheon 24410, Republic of Korea; 6 Department of Integrative Medicine, Major in Digital Healthcare, Yonsei University College of Medicine, Seoul 06229, Republic of Korea

**Keywords:** biomass, chitosan, humidity sensor, molecular dynamics

## Abstract

This study presents the fabrication of a sustainable flexible humidity sensor utilizing chitosan derived from mealworm biomass as the primary sensing material. The chitosan-based humidity sensor was fabricated by casting chitosan and polyvinyl alcohol (PVA) films with interdigitated copper electrodes, forming a laminate composite suitable for real-time, resistive-type humidity detection. Comprehensive characterization of the chitosan film was performed using Fourier-transform infrared (FTIR) spectroscopy, contact angle measurements, and tensile testing, which confirmed its chemical structure, wettability, and mechanical stability. The developed sensor exhibited a broad range of measurements from 6% to 97% relative humidity (RH), a high sensitivity of 2.43 kΩ/%RH, and a rapid response time of 18.22 s with a corresponding recovery time of 22.39 s. Moreover, the chitosan-based humidity sensor also demonstrated high selectivity for water vapor when tested against various volatile organic compounds (VOCs). The superior performance of the sensor is attributed to the structural properties of chitosan, particularly its ability to form reversible hydrogen bonds with water molecules. This mechanism was further elucidated through molecular dynamics simulations, revealing that the conductivity in the sensor is modulated by proton mobility, which operates via the Grotthuss mechanism under high-humidity and the packed-acid mechanism under low-humidity conditions. Additionally, the chitosan-based humidity sensor was further seamlessly integrated into an Internet of Things (IoT) framework, enabling wireless humidity monitoring and real-time data visualization on a mobile device. Comparative analysis with existing polymer-based resistive-type sensors further highlighted the superior sensing range, rapid dynamic response, and environmental sustainability of the developed sensor. This eco-friendly, biomass-derived, eco-friendly sensor shows potential for applications in environmental monitoring, smart agriculture, and industrial process control.

## 1. Introduction

Environmental factors, such as temperature, pressure, and humidity play a critical role in various domains, necessitating precise measurement and regulation. Among these, humidity stands out as a critical parameter influencing diverse sectors such as industry, agriculture, medicine, food preservation, human comfort, and environmental monitoring [1,2,3,4]. Accurate detection of relative humidity, which defines the moisture content in the atmosphere, is crucial even at minimal levels and requires advanced, reliable instrumentation [1,5]. To monitor changes in humidity, various sensing mechanisms based on resistive [6], capacitive [7], impeditive [8], acoustic [9], and optical [10] methods have been developed, each offering unique advantages for specific applications. Consequently, humidity sensors have garnered substantial research interest due to their indispensable role in maintaining optimal environmental conditions. The ideal humidity sensor is characterized by attributes such as rapid response and recovery times, long-term stability, high precision, and seamless integration with modern devices and systems [5].

Furthermore, desirable properties include high sensitivity, minimal hysteresis, and cost-effectiveness, making them suitable for widespread adoption across diverse applications. A wide range of humidity sensors has been developed using diverse materials, such as ceramics [11], polymers [12], and carbon-based materials [13,14], tailored to different sensing mechanisms.

Among these, polymer-based sensors, in particular, have garnered substantial interest owing to their lightweight nature, ease of fabrication, flexibility, and adaptability to various applications [15,16]. Recent advancements have further spurred interest in natural polymers for sensor development, driven by their non-toxicity, biocompatibility, chemical inertness, and environmental sustainability.

For instance, Li et al. reported a humidity sensor created by coating silk fabric with a graphene oxide sensing layer using successive electroless plating of conductive interdigital electrodes [17], which demonstrated excellent humidity-sensing capabilities. Similarly, Wang et al. developed an innovative sensor integrating PVA/MXene nanofibers and a monolayer MoSe_2_ piezoelectric nanogenerator, achieving high-performance humidity monitoring alongside energy harvesting for wearable devices [18]. Additionally, Soomro et al. introduced a biocompatible humidity sensor characterized by fast, linear, and all-range sensitivity. This sensor, employing polylactic glycolic acid (PLGA) as a sensing layer, demonstrates suitability for various applications [19].

Recent advancements in humidity sensor technology have increasingly focused on natural polymers such as chitosan, which is renowned for its strong affinity toward water molecules due to the presence of hydrophilic functional groups including hydroxyl and amine. This inherent property has catalyzed extensive research into chitosan-based sensors and their modifications for enhanced water molecule adsorption [2,20,21,22]. For instance, Liu et al. developed a high-performance humidity sensor employing a chitosan/polypyrrole composite in a quartz crystal microbalance configuration, enabling precise respiratory pattern monitoring through a dedicated application [22]. Similarly, Kumari et al. fabricated a resistive-type humidity sensor employing chitosan films, demonstrating significant potential for human health monitoring [2]. Zou et al. further explored chitosan’s capabilities by introducing a protonic conductive sensor that exhibited a substantial conductance response to fluctuations in humidity levels [21]. These developments highlight the evolving landscape of polymer-based humidity sensor technology, with chitosan emerging as a promising material due to its superior functionality, sensitivity, and versatility in diverse applications.

Conventional humidity sensors often rely on pristine substrate materials, which can miss the potential for more sustainable and eco-friendly alternatives. This reliance has sparked investigations into alternative methods, driving innovation in sensor design and fabrication to address material and environmental limitations. The increasing demand for sustainable technologies underscores the need to explore alternative material sources for the fabrication of polymer-based humidity sensors. Among potential sources, mealworms have emerged as a promising candidate due to their suitability for large-scale production, adaptable biological requirements, and the wealth of existing knowledge facilitating effective commercial production [23]. However, the rapid expansion of mealworm farming has resulted in significant biomass waste generation, raising environmental concerns. This biomass, primarily composed of chitin-rich mealworm shells, offers a valuable opportunity as a raw material for chitosan extraction. Chitosan, derived through the deacetylation of chitin, is a versatile biopolymer characterized by its biodegradability, biocompatibility, and non-toxic properties [24]. These attributes make it particularly well-suited for sensing applications. Consequently, utilizing chitosan extracted from biowaste not only mitigates environmental challenges associated with waste management but also provides a cost-effective and sustainable alternative for humidity sensor fabrication. By leveraging the inherent hydrophilic properties and chemical versatility of chitosan, this approach advances the development of environmentally friendly sensing technologies while aligning with global efforts toward sustainability in material science and sensor innovation.

In this study, we develop a flexible, resistive-type humidity sensor utilizing chitosan film derived from biomass and polyvinyl alcohol (PVA) as key materials, coupled with copper interdigitated electrodes (IDEs), to achieve high-performance sensing capabilities. Chitosan, extracted from mealworm biomass, serves as the primary sensing layer, due to its exceptional hydrophilic properties and compatibility with water vapor adsorption. A layer of copper was precisely patterned on the chitosan film to form IDEs, and PVA was subsequently applied via drop casting, resulting in a robust, laminate composite structure, forming the complete chitosan-based humidity sensor. Comprehensive characterization techniques, including chemical structure analysis and surface properties evaluations, were employed to validate the sensor’s design and functionality. The fabricated sensor demonstrated outstanding performance metrics, specifically high sensitivity, minimal hysteresis, rapid response and recovery times, excellent reversibility, a broad detection range, and strong selectivity. To elucidate the underlying sensing mechanism, molecular dynamics simulations were performed, offering further insights into the sensor’s operation at a molecular level. This dual approach of experimental and computational analysis underscores the sensor’s potential for practical applications. Additionally, the sensor’s capability for wireless humidity sensing was validated through its integration into an Internet of Things (IoT) framework, enabling real-time monitoring and data visualization. This approach innovatively utilizes biowaste resources, providing dual benefits by addressing environmental challenges and advancing sustainable humidity sensor technology. By utilizing readily available raw materials and employing a straightforward fabrication process, the proposed design ensures feasibility for large-scale production, combining practicality with sustained performance.

The structure of the manuscript is as follows. Section 1 introduces the significance of humidity sensors and highlights the role of chitosan as a sustainable sensing material. Section 2 outlines the detailed processing and fabrication methodologies for the chitosan-based humidity sensor. Section 3 presents the experimental results, including performance evaluations and discussions, supported by molecular dynamics simulations. Finally, Section 4 summarizes the findings and discusses broader applications of the proposed sensor.

## 2. Materials and Methods

### 2.1. Materials

All chemicals and reagents used in this study were of analytical grade to ensure high precision and reliability in experimental outcomes.

Sodium hydroxide (NaOH) was procured from OCI Company Ltd., Seoul, Republic of Korea. Dimethyl sulfoxide (DMSO, (CH_3_)_2_SO), acetic acid (CH_3_COOH), lithium bromide (LiBr), potassium acetate (CH_3_COOK), magnesium chloride (MgCl_2_), potassium carbonate (K_2_CO_3_), magnesium nitrate (Mg(NO_3_)_2_), sodium chloride (NaCl), potassium chloride (KCl), potassium sulfate (K_2_SO_4_), acetone (C_3_H_6_O), ethanol (C_2_H_5_OH), isopropanol (C_3_H_7_OH), and methanol CH_3_OH were procured from DAEJUNG Reagents Chemicals and Metals Co., Ltd., Siheung, Republic of Korea. Polyvinyl alcohol (PVA, (C_2_H_4_O)x) was obtained from SAMCHUN Chemicals Co., Ltd., Seoul, Republic of Korea. For filtration and other processing needs, 475855-1R Miracloth was sourced from EMD Millipore Corp., Billerica, MA, USA. All materials were handled and stored in accordance with their respective safety and usage guidelines to maintain consistency and safety throughout the study.

#### Source of Biomass

The Gangwon Provincial Agricultural Products Center, located in Chuncheon, Republic of Korea, operates a state-of-the-art smart farming facility dedicated to the large-scale production of yellow mealworm beetles (*Tenebrio molitor*). This facility employs cutting-edge artificial intelligence and IoT systems to meticulously monitor and optimize environmental parameters such as temperature, humidity, and feeding cycles, ensuring optimal growth and health of the mealworms. Through the integration of robust genetic assessment and selective breeding techniques, the facility prioritizes high reproductive rates, accelerated development, and enhanced survival, achieving an annual production of approximately 10 tons of mealworms. The significant biomass generated during this process, primarily consisting of mealworm shells, represents a renewable and underutilized resource. This surplus biomass, otherwise a byproduct of mealworm farming, is harnessed in our study for the extraction of chitosan, demonstrating a sustainable approach to material sourcing. By utilizing this biowaste, the study aligns with global efforts to promote environmental sustainability and circular economy principles in scientific innovation.

### 2.2. Extraction of Chitosan

**Figure 1:**
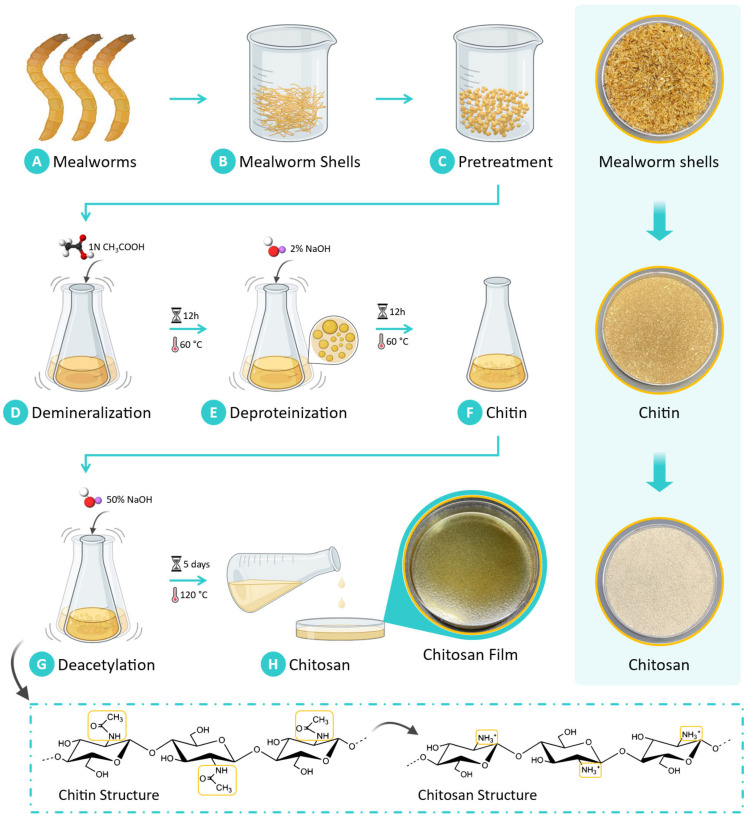
The sequential steps involved in extracting chitosan from mealworm shells. (**A**) Mealworms cartoon depiction. (**B**) Mealworm shells are obtained. (**C**) These flakes are finely pulverized into a powder form during pretreatment. (**D**) The purification process begins with demineralization, wherein the powder is dissolved in acetic acid solution to remove minerals present in the biomass. (**E**) Deproteinization is carried out using an alkali digestion method, wherein a sodium hydroxide solution is employed to remove protein content from the material. The proteins are associated with the chitin structure through glycosidic bonds. (**F**) This step isolates chitin as the primary product. (**G**) Chitosan is then derived from chitin via a deacetylation process, which involves treating chitin with a concentrated sodium hydroxide solution. During this reaction, the N-acetyl groups within the chitin are transformed into amino groups, yielding chitosan. (**H**) The resulting chitosan is subsequently cast onto a petri dish for further use. Digital photos of the resulting chitosan film are shown above along with the obtained mealworm shells (biomass), the extracted chitin, and the synthesized chitosan (far right).

The extraction of chitosan from *Tenebrio molitor* larvae biomass was conducted following the methodology previously reported by our group [25]. Figure 1 illustrates the chitosan extraction process. This process involves demineralization using acetic acid, deproteinization with sodium hydroxide, and subsequent deacetylation to convert chitin to chitosan. In brief, mealworm shells are collected, washed with hot water, and dried to form flakes, which are ground into a powder. In the demineralization phase, the powder is treated with a 1 N acetic acid solution at 60 °C for 12 h to remove mineral components, followed by rinsing and drying. Next, the deproteinization step removes protein by treating the demineralized material with a 2% sodium hydroxide solution at 60 °C for 12 h. The final phase, deacetylation, involves treating the purified chitin with 50% sodium hydroxide at 120 °C for five days to convert it to chitosan. The chitosan is then filtered, washed, and purified. This method aligns with established protocols for chitosan extraction.

### 2.3. FTIR Analysis, Contact Angle Measurement, and Film Thickness Evaluation

The chemical composition of the chitosan film derived from biomass was characterized through Fourier-transform infrared (FTIR) spectroscopy using an Alpha II spectrometer (Bruker, Bremen, Germany). The FTIR spectra provided detailed insights into the molecular structure of the extracted chitosan, enabling confirmation of its chemical profile and the presence of characteristic functional groups.

To assess the surface wettability of the chitosan film, contact angle measurements were performed. A 3 μL droplet of deionized water was carefully deposited onto a 3 × 3 cm^2^ section of the film using a micropipette. The contact angle was determined as the mean value of three replicate measurements taken at different points on the sample to ensure reliability and accuracy. The contact angle provided insights into the hydrophilic or hydrophobic nature of the film, a critical parameter for assessing its interaction with water vapor. Additionally, the thickness, a crucial factor influencing the sensor’s performance, was measured using a digital vernier caliper (model DC150-1, CAS Co. Ltd., Yangju, Republic of Korea). Five measurements were taken at random points on each film sample, and the mean value was calculated and reported as the final thickness.

### 2.4. Fabrication of the Biomass-Derived Chitosan-Based Humidity Sensor

The fabrication of the chitosan-based humidity sensor followed a meticulously designed process to ensure precision, reproducibility, and optimal performance. Initially, chitosan derived from biomass was cast into thin, uniform films, serving as the primary sensing layer. The copper tape was then applied to the chitosan film to act as a conductive material for the electrode configuration. To achieve an interdigitated electrode (IDE) pattern with high resolution, the copper tape was cut using a roll-through cutter (Portrait 3, Silhouette America Inc., Lindon, UT, USA). This step allowed for precise electrode patterning directly on the chitosan film, essential for effective humidity sensing. In parallel, a solution of polyvinyl alcohol (PVA) was prepared by dissolving the polymer in dimethyl sulfoxide (DMSO) to achieve 20% (*w*/*v*) concentration.

**Figure 2:**
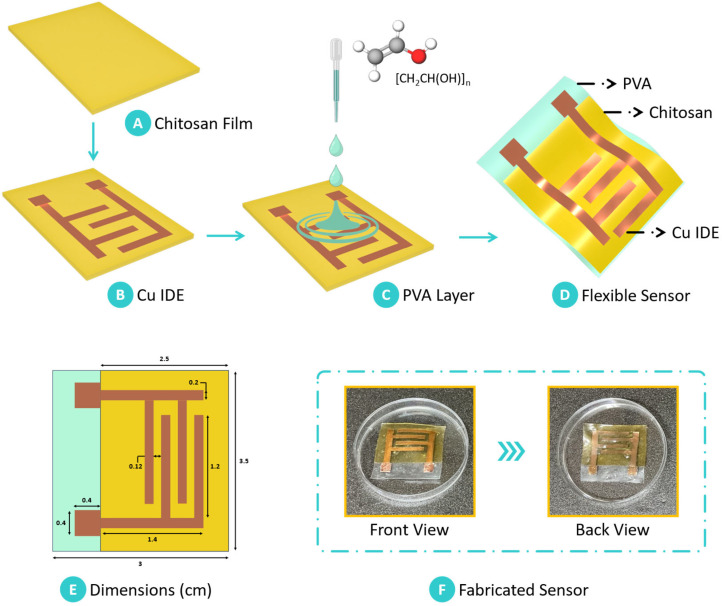
Step-by-step procedure for fabricating the biomass-derived chitosan-based humidity sensor. (**A**) The biomass-derived chitosan film serves as the primary sensing material. (**B**) Copper tape is affixed to the chitosan film, and an interdigitated electrode (IDE) pattern is precisely cut. (**C**) Polyvinyl alcohol (PVA) solution is drop-cast onto the chitosan film with the IDE pattern, (**D**) resulting in the formation of the laminate composite chitosan-based humidity sensor. (**E**) Dimensions of the developed sensor are shown, along with (**F**) a digital photograph displaying the front and back views of the final humidity sensor assembly.

The mixture was stirred for two hours at 60 °C to ensure complete dissolution, resulting in a homogeneous PVA solution. This PVA solution was then carefully drop-cast onto the chitosan film layered with the copper IDE, creating a laminate composite structure. DMSO was selected due to its high solvency for PVA, which ensured a homogeneous and consistent solution. While water and ethanol were considered as potential solvents, they did not achieve the required solubility, which is crucial for uniform film formation. The use of DMSO contributed to the fabrication of high-quality films with optimal performance characteristics. The addition of PVA provided a substrate layer that enhanced the structural stability of the chitosan–copper assembly while retaining flexibility. The resulting multilayer structure comprised the chitosan film as the sensing layer, copper electrodes as the conductive element, and PVA as a stabilizing substrate. This composite design facilitated high sensitivity and robustness in detecting humidity changes. Figure 2 provides a schematic representation of the fabrication process, detailing each stage in the assembly of the biomass-derived chitosan-based humidity sensor.

### 2.5. Humidity Response Measurement

The humidity sensitivity of the chitosan-based sensor was evaluated in controlled environments with precisely regulated relative humidity (RH) levels. Saturated salt solutions were employed to generate specific RH conditions, as they are cost-effective, widely used, and capable of maintaining consistent humidity levels in enclosed systems. These saturated solutions were prepared using various salts, each corresponding to a defined RH level: lithium bromide (LiBr, RH: 6%), potassium acetate (CH_3_COOK, RH: 23%), magnesium chloride (MgCl_2_, RH: 33%), potassium carbonate (K_2_CO_3_, RH: 43%), magnesium nitrate (Mg(NO_3_)_2_, RH: 53%), sodium chloride (NaCl, RH: 75%), potassium chloride (KCl, RH: 84%), and potassium sulfate (K_2_SO_4_, RH: 97%). The preparation of these solutions involved dissolving the salts in deionized water until saturation was achieved. The solutions were then placed in sealed containers to create stable RH conditions for sensor testing. The sensor was exposed to each RH level sequentially, and its electrical resistance was monitored to measure its responsiveness to changes in humidity. This approach enabled the generation of a broad spectrum of RH conditions, facilitating a comprehensive evaluation of the sensor’s performance across the entire detection range. The use of saturated salt solutions ensured accuracy and reproducibility in assessing the sensor’s humidity response.

**Figure 3:**
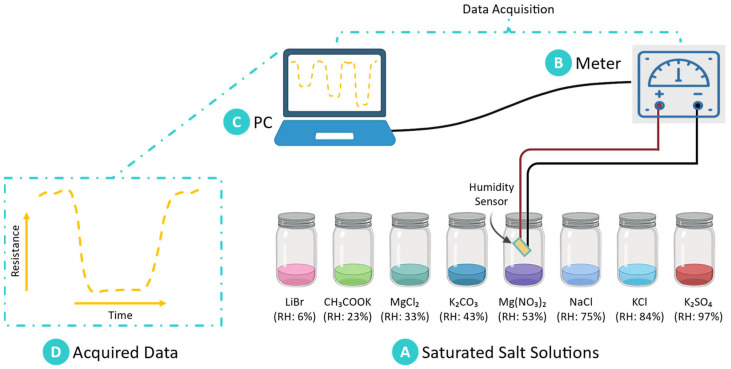
Schematic representation of the experimental setup for evaluating humidity-sensing performance. (**A**) Controlled relative humidity (RH) levels are established using saturated salt solutions placed in sealed containers to create stable testing environments. (**B**) A Keithley source meter is utilized to apply voltage and measure the resistance of the chitosan-based humidity sensor in real-time. (**C**) Data acquisition and analysis are conducted via a connected PC, enabling continuous monitoring of sensor responses. (**D**) Sample data showing resistance change over time as humidity varies.

An experimental setup was meticulously designed to ensure stable and accurate RH conditions for sensor testing, as illustrated in Figure 3. The setup consisted of sealed containers housing saturated salt solutions, which allowed us to maintain constant humidity levels around the sensor. To mitigate fluctuations caused by temperature variations, a water bath was integrated into the system, maintaining a constant temperature of 25 °C throughout the testing period. This temperature regulation was essential for ensuring the consistency and reliability of RH readings. During each test, the dynamic response of the chitosan-based sensor to varying RH levels was systematically recorded. Resistance changes in the sensor were monitored at consistent intervals to capture its responsiveness across the full range of RH conditions. A Keithley source meter was employed to measure the sensor’s electrical response, applying a constant voltage of 1 V DC. The conductance of the sensor was carefully recorded for each RH level. This methodology facilitated precise assessment of the sensor’s sensitivity, stability, and responsiveness under controlled conditions.

### 2.6. Molecular Modeling and Simulation

**Figure 4:**
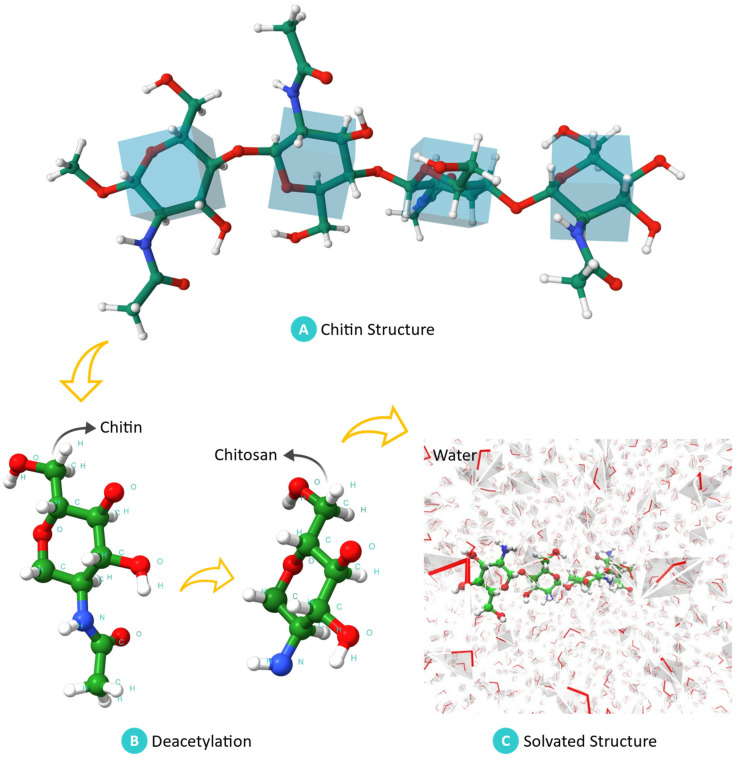
Molecular dynamics simulation illustrating the structural evolution of solvated chitosan. (**A**) The modeled chitin structure with a composition of four units of β-1,4-linked N-acetylglucosamine along with a methyl ester saturation. The carbon (C) atoms are denoted in green, oxygen (O) atoms in red, hydrogen (H) atoms in white, and nitrogen (N) atoms in blue. (**B**) The transformation of chitin to chitosan during the deacetylation process, depicted by the removal of N-acetyl groups, reveals the emergence of amino groups critical for humidity-sensing applications. (**C**) A snapshot of the simulation cell. Notably, the resulting chitosan structure is entirely solvated by water molecules within a cubic box, with a designated offset of 10 Å to ensure comprehensive solvation. This solvation process incorporates a total of 2626 water molecules to fully immerse the chitosan structure, as depicted in the simulation snapshot.

The interaction between chitosan and water molecules was investigated through molecular simulations to gain insight into the structural behavior of chitosan in aqueous environments. Molecular dynamics (MD) simulations were conducted using the Large-scale Atomic/Molecular Massively Parallel Simulator (LAMMPS) package [26], a powerful tool for modeling complex molecular systems. The simulations were run on a PC equipped with an AMD Ryzen 5 5600 6-core processor (AMD, Addison, TX, USA) operating at 3.50 GHz. Initially, the molecular modeling of the chitin structure was carried out using the Carbohydrate Builder of GlyCam [27], an online tool specialized in carbohydrate modeling. This process generated a chitin structure composed of four units of β-1,4-linked N-acetylglucosamine, as depicted in Figure 4A. Subsequently, the chitin structure was chemically modified to obtain the chitosan structure by removing the acetyl group from the molecular chain, as illustrated in Figure 4B. The deacetylation process, which converted chitin to chitosan, was achieved using PyMol software (Version 3.1, Shrodinger, New York, NY, USA) [28], ensuring a degree of deacetylation of 100%. To prepare the chitosan structure for MD simulations, solvation was performed using the CHARMM-GUI interface [29,30]. The chitosan structure was solvated with 2626 water molecules employing the TIP3P water model, with a solvation edge distance of 10 Å.

The resulting solvated chitosan structure, depicted in Figure 4C, was used as the basis for subsequent MD simulations conducted with LAMMPS software (https://www.lammps.org, access date: 31 August 2021). The simulation cell was configured as a cubic box with dimensions of 44 Å per side, providing an appropriate environment for studying the molecular interactions. This simulation setup allowed for the detailed examination of the molecular interactions between chitosan and water, providing critical insights into hydrogen bonding, structural stability, and the hydration dynamics essential for understanding chitosan’s behavior in humidity-sensing applications.

The energy minimization, equilibration, and dynamics of the chitosan structure were further conducted using LAMMPS. Throughout these simulations, the interatomic interactions were determined employing the CHARMM36m force field [31], which is renowned for its accuracy in representing atomic interactions in polysaccharides [32,33,34,35]. The choice of a suitable force field was critical to achieving reliable simulation outcomes, as it provides a robust framework for capturing the structural and dynamic properties of biomolecules.

Long-range electrostatic interactions were handled using the particle–particle–particle-mesh (PPPM) solver [36], an efficient method for maintaining computational accuracy over extended distances. Periodic boundary conditions (PBCs) were implemented in all directions of the simulation cell to simulate an infinite system and eliminate edge effects, ensuring continuity and realism in the simulated environment.

The simulation process began with energy minimization to stabilize the system and eliminate steric clashes. This step was performed using the conjugate gradient method, which efficiently optimized the system’s energy state. Following energy minimization, the system underwent equilibration in two distinct thermodynamic ensembles: the NVT ensemble (constant number of particles, volume, and temperature) and the NPT ensemble (constant number of particles, pressure, and temperature). These steps were essential to achieving thermal and pressure equilibrium, ensuring the stability of the solvated chitosan structure. The combination of these processes established a well-equilibrated system, suitable for subsequent analysis of the chitosan–water interactions and their implications for humidity-sensing applications.

To ensure the structural stability and accuracy of the simulations, all hydrogen-containing bonds within the chitosan structure were constrained using the SHAKE algorithm [37]. The integration of Newton’s equations of motion was achieved through the velocity-Verlet scheme, utilizing a timestep of 1 fs to capture intricate molecular movements accurately. Equilibration within the NVT ensemble was conducted at 298 K, while in the NPT ensemble, equilibration was achieved at 298 K and 1 atm. Constant temperature and pressure were maintained throughout the simulations by employing the Nosé–Hoover thermostat and barostat, respectively. For visualization and in-depth analysis of the chitosan structure, UCSF Chimera software (Version 1.16) [38] and VMD software (Version 194a) [39] were utilized. These tools provided comprehensive insights into the molecular configuration and dynamics of the chitosan structure. For the hydrogen bond analysis, the atomic distance between the donor and acceptor atoms involved in hydrogen bonds was defined as 3.2 Å with an angle of 135°. To investigate the hydration behavior of chitosan, computations were performed to quantify the number of hydrogen bonds and the solvent-accessible surface area (SASA). These criteria ensured the accurate identification of hydrogen bonding interactions. The SASA calculations provided a quantitative measure of the extent to which water molecules interacted with the chitosan structure, while hydrogen bond analysis revealed the nature and strength of these interactions.

## 3. Results and Discussion

### 3.1. Characterization and Testing of the Biomass-Derived Chitosan Film

**Figure 5:**
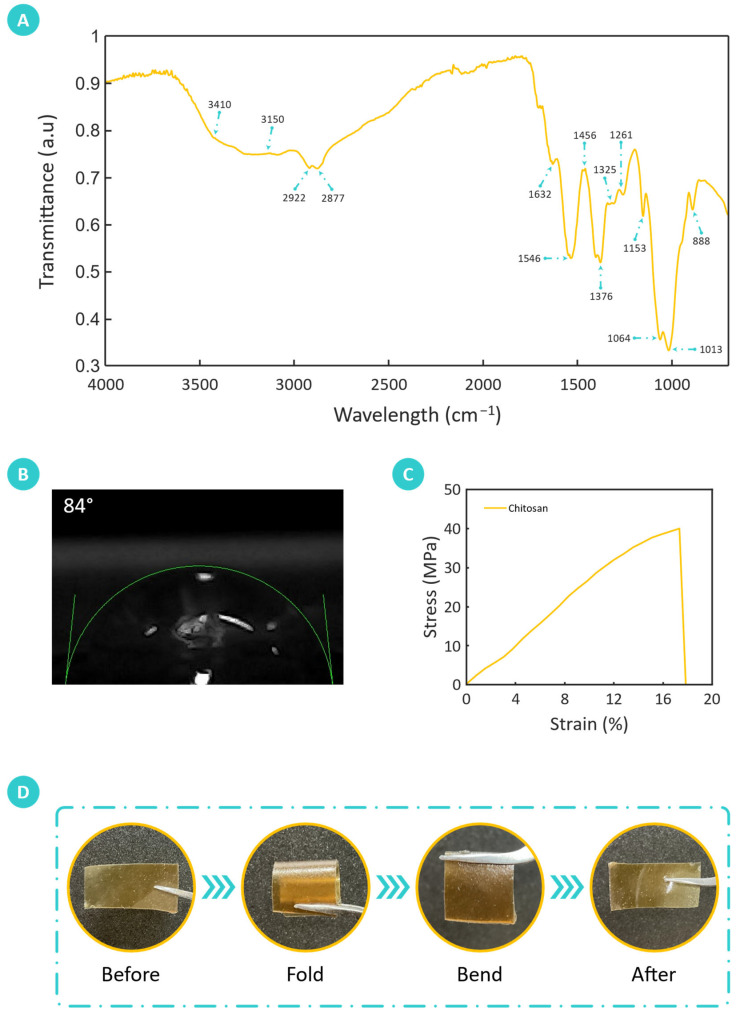
Comprehensive characterization of the fabricated chitosan film. (**A**) Fourier-transform infrared (FTIR) spectroscopy analysis elucidates the chemical structure and composition of the biomass-derived chitosan. (**B**) Contact angle measurement shows a contact angle of 84 ± 1.58°, indicating moderate wettability. (**C**) Stress–strain analysis demonstrates the mechanical robustness of the film, with an ultimate tensile strength (UTS) of 39.92 MPa and an elongation at break (EAB) of 17.49%, reflecting a balance of strength and flexibility. (**D**) Flexibility assessment of the film through deformation tests, demonstrating its ability to bend and fold without structural failure.

Fourier-transform infrared (FTIR) spectroscopy was utilized to analyze the molecular properties of chitosan derived from biomass, providing a detailed assessment of its chemical structure. Figure 5A presents the FTIR spectrum of the extracted chitosan, confirming the molecular composition and structural integrity of the extracted chitosan. Notably, the broadened peak spanning 3410 cm^−1^ to 3150 cm^−1^ signifies robust stretching of N–H and O–H groups, indicating abundant amine and hydroxyl hydrophilic functional groups. These hydrophilic functional groups are known to exhibit a strong affinity toward water molecules, highlighting the potential of biomass-extracted chitosan for humidity-sensing applications. Moreover, the absorptive features observed around 2922 cm^−1^ and 2877 cm^−1^ correspond to symmetric and asymmetric C–H bond stretching and are consistent with the polysaccharide backbone of chitosan. The presence of residual N-acetyl groups, amide I and amide II, is evidenced by the peaks at 1632 cm^−1^ and 1325 cm^−1^, respectively, further confirming the chitosan’s molecular structure.

Additionally, peaks at 1546 cm^−1^ are attributed to the bending vibration of the primary amine (N–H) group [40], a key feature of chitosan’s chemical structure. Peaks at 1456 cm^−1^ (CH_2_ bending) and 1376 cm^−1^ (CH_3_ symmetrical deformations) provide further insights into the molecular vibrations within the chitosan sample. The distinctive signal at 1261 cm^−1^ is attributed to the OH bending vibrations, further confirming the presence of hydrophilic groups inherent to chitosan [41]. Furthermore, the absence of strong signals in the 1260–1270 cm^−1^ region effectively dismisses the occurrence of glycosaminoglycan (GAG) contamination in the extracted chitosan material, affirming its purity. The peak observed at 1153 cm^−1^ indicates C–O–C bridge asymmetric stretching, while the peaks at 1064 cm^−1^ and 1013 cm^−1^ are associated with the stretching of C–O. Furthermore, the peak observed at 888 cm^−1^ corresponds to out-of-plane CH bending of the monosaccharide ring structure [42], emphasizing the molecular arrangement’s integrity.

Collectively, these findings confirm the chemical integrity and high purity of the fabricated chitosan film. The presence of well-defined functional groups and a robust polysaccharide backbone highlights the material’s potential for humidity-sensing applications. Its molecular structure, characterized by hydrophilic groups and stable chemical bonds, plays a pivotal role in water vapor adsorption and detection. Moreover, the measured FTIR spectrum aligns closely with previously reported spectra of chitosan [41,42,43], further validating the reliability and consistency of the extracted material.

The contact angle of the fabricated chitosan film was 84 ± 1.58°, as shown in Figure 5B. Contact angle measurement holds significance in humidity sensing, demonstrating the wettability and other attributes inferred from this value. A contact angle of 84 ± 1.58° indicates moderate hydrophilicity of the chitosan film, suggesting that it possesses a certain degree of affinity for water molecules while also exhibiting some hydrophobic characteristics. In the context of humidity sensing, this moderate hydrophilicity implies that the chitosan film can readily adsorb water vapor from the surrounding environment, making it suitable for detecting changes in humidity levels. The moderate contact angle value suggests that the chitosan film strikes a balance between hydrophilicity and hydrophobicity, which is advantageous for humidity-sensing applications. A hydrophilic surface allows for efficient adsorption of water molecules, enabling the chitosan film to accurately detect variations in humidity levels. On the other hand, the presence of some hydrophobic properties can prevent excessive water absorption, preventing swelling and ensuring the stability and durability of the sensor over time.

The mechanical properties of the fabricated chitosan film were comprehensively evaluated using a tensile testing machine, to assess its durability and adaptability under mechanical stress. The measured thickness of the fabricated chitosan film was 0.23 ± 0.02 mm. The stress–strain plot shown in Figure 5C shows the film’s mechanical response, having an ultimate tensile strength (UTS) of 39.92 MPa and a corresponding elongation at break (EAB) value of 17.49%. These values indicate that the chitosan film possesses both sufficient strength and moderate ductility. The UTS of 39.92 MPa suggests that the film can withstand substantial tensile loads, making it durable enough to maintain its structural integrity during extended use in humidity-sensing applications. Meanwhile, an EAB of 17.49% demonstrates a balance between rigidity and flexibility, which is advantageous for a sensing material that may undergo repeated bending and stretching in practical applications. This balance supports its potential for integration into flexible electronic systems and contributes to its suitability for dynamic sensing environments, where mechanical stability and flexibility are essential.

Flexibility is a desirable attribute for sensors, as it enables the sensor to conform to various shapes and surfaces, enhancing its versatility and applicability in different environments and applications. As shown in Figure 5D, the fabricated chitosan film demonstrated excellent flexibility, enabling it to be folded and bent without compromising its structural integrity. This characteristic is particularly advantageous for humidity sensing, where adaptability to complex and irregular surfaces is often required. The flexibility of the chitosan film ensures that it can adapt to the contours of the substrate or surface onto which it is applied. This flexibility facilitates intimate contact between the sensor and the surrounding environment, promoting efficient interaction with water vapor molecules.

By conforming to the surface morphology, the chitosan film maximizes its available surface area for water vapor adsorption, thereby improving the sensitivity and responsiveness of the biomass-derived chitosan-based humidity sensor to changes in humidity levels. Additionally, the flexibility of the chitosan film contributes to the mechanical robustness and durability of the sensor. The ability of the film to withstand repeated deformations without structural damage ensures long-term reliability and performance stability, even under harsh operating conditions. Moreover, the flexibility of the chitosan film allows for easy integration into complex structures or devices, enabling the development of miniaturized and portable humidity-sensing systems. The ability to fold or bend the sensing material without compromising its integrity enhances its versatility and usability in various applications, including wearable devices, IoT sensors, and biomedical instruments.

### 3.2. Performance Assessment of the Biomass-Derived Chitosan-Based Humidity Sensor

#### 3.2.1. Dynamic Humidity Response

**Figure 6:**
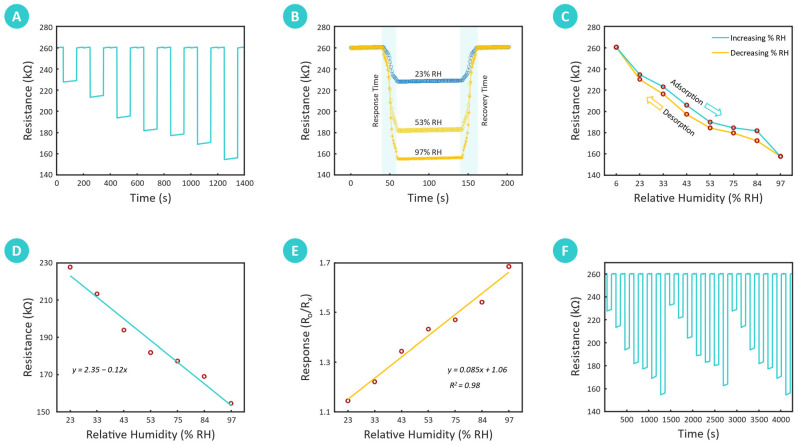
Performance evaluation of the chitosan-based humidity sensor. (**A**) Dynamic response of the chitosan-based humidity sensor to varying relative humidity levels. The graph depicts the resistance of the sensor as a function of time, showcasing its real-time adaptability as the humidity transitions from 6% to 97%. This temporal profile highlights the sensor’s ability to dynamically adjust its resistance based on moisture adsorption and desorption processes, a key attribute for applications requiring continuous monitoring of environmental humidity. (**B**) Response and recovery characteristics, with an average response time of 18.22 s and an average recovery time of 22.39 s, showcasing fast adsorption and desorption rates. (**C**) Hysteresis analysis of the sensor, quantified at 9.04% at 53% RH, confirmed the sensor’s reliability in responding to RH changes. (**D**) A linear decrease in resistance with increasing RH shows a strong correlation with a high R^2^ value. (**E**) Sensor response (Ro/Rx) as a function of RH, with a high correlation coefficient (R^2^ = 0.98), indicating reliable linear performance. (**F**) Reproducibility and reversibility assessment, illustrating the sensor’s consistent response across multiple cycles and RH levels, demonstrates its stability for long-term application.

The dynamic humidity response of the biomass-derived chitosan-based humidity sensor was evaluated by measuring resistance variations across a wide range of relative humidity (RH) conditions, established with saturated salt solutions. As shown in Figure 6A, the sensor’s resistance consistently decreased with increasing RH, demonstrating its capability to effectively detect and respond to humidity changes. Specifically, the resistance dropped from 260.23 kΩ at 6% RH to progressively lower values at higher RH levels, with measured resistances of 228.37 kΩ at 23% RH, 214.17 kΩ at 33% RH, 194.74 kΩ at 43% RH, 182.60 kΩ at 53% RH, 177.93 kΩ at 75% RH, 169.93 kΩ at 84% RH, and reaching a minimum of 155.42 kΩ at 97% RH. This clear inverse relationship between resistance and RH illustrates the high sensitivity of the developed sensor to different levels of relative humidity. The observed decrease in resistance is attributed to the increased adsorption of water molecules onto the chitosan film. Chitosan, with its hydrophilic functional groups such as hydroxyl and amine groups, interacts with water molecules through hydrogen bonding, leading to enhanced ionic conductivity. As the RH increases, more water molecules are adsorbed, forming a continuous conductive pathway that facilitates ionic conduction.

#### 3.2.2. Response–Recovery Rate, Sensitivity, and Hysteresis

The response and recovery dynamics of the biomass-derived chitosan-based humidity sensor, as depicted in Figure 6B, further underscore its rapid responsiveness to fluctuating humidity levels. The response time, defined as the required time interval for the sensor’s resistance to change by 90% of its peak value upon exposure to increased RH, was found to be 18.22 s. Similarly, the recovery time, which represents the interval for the resistance to return to baseline upon RH reduction, was 22.39 s.

These rapid response and recovery characteristics emphasize the sensor’s ability to swiftly adapt to changes in ambient moisture levels. The observed fast response times can be attributed to the highly hydrophilic nature of chitosan, derived from its abundant hydroxyl and amine functional groups. These groups facilitate rapid adsorption of water molecules from the environment during humidification and efficient desorption during dehumidification. The molecular interaction between the chitosan and water molecules ensures quick ionic conduction adjustments within the sensing layer, thereby enabling real-time resistance changes corresponding to humidity variations.

This rapid response behavior is advantageous for real-time monitoring applications, where quick adaptation to environmental changes is essential.

Sensitivity is another crucial parameter, reflecting the sensor’s ability to detect incremental changes in *RH*. Sensitivity (*S*) was calculated as the change in resistance per unit change in RH using Equation (1):

(1) $$S=\frac{\left(R_{o\;}-R_{x}\right)}{{RH}_{o\;}-{RH}_{x}}$$

where *S* is the sensitivity in Ω/%RH, *Ro* represents the resistance at the lowest humidity level (6% RH), and *Rx* represents the resistance at other RH values. The sensor’s average sensitivity was determined to be 1.89 kΩ/%RH, with a peak sensitivity of 2.43 kΩ/%RH. This high sensitivity indicates the chitosan film’s strong responsiveness to even minor RH variations, enhancing its utility in applications requiring precise humidity control.

(2) H(%)=Ra −RdS×100

where *R_a_* and *R_d_* represent resistance values during adsorption (humidification) and desorption (dehumidification), respectively. A low hysteresis value, as observed here, indicates minimal discrepancies between resistance values during the humidification and dehumidification cycles. This low hysteresis is beneficial for practical applications, as it implies that the sensor can reliably reproduce consistent readings across changing RH conditions, with minimal lag or delay in response.

The low hysteresis enhances the sensor’s precision, ensuring stable performance for continuous monitoring applications. This characteristic is particularly useful in fields that require precise humidity control, including environmental monitoring, industrial process control, and healthcare. Consistent readings, combined with high sensitivity and rapid response–recovery rates, demonstrate the effectiveness of the developed biomass-derived chitosan-based humidity sensor in delivering reliable, real-time data, making it a promising sensing material for advanced humidity-sensing technologies. The low hysteresis exhibited by the chitosan-based humidity sensor is particularly advantageous for practical applications, as it ensures accurate and repeatable measurements during cyclic humidity transitions. This characteristic minimizes lag and signal distortion, enabling the sensor to maintain consistent performance even in dynamic monitoring scenarios. Applications such as environmental monitoring, industrial process control, and agricultural systems benefit greatly from sensors with low hysteresis, as these systems often require real-time adjustments based on fluctuating humidity levels. The observed low hysteresis value of the sensor further underscores its reliability and suitability for such demanding applications.

#### 3.2.3. Humidity Response Correlation

The relationship between the biowaste-derived chitosan-based humidity sensor’s resistance and its response at varying levels of relative humidity is shown in Figure 6D,E. These graphs provide insight into how the sensor’s resistance varies systematically with RH and the corresponding response—defined as *R_o_*/*R_x_*, where *R_o_* is the resistance at 6% RH and *R_x_* is the resistance at each successive RH level. As seen in Figure 6D, the measured resistance decreases linearly with increasing RH, a trend that reflects the increased conductivity of the chitosan film as it adsorbs moisture. The linear correlation between resistance and RH supports the sensor’s potential for precise monitoring, as it enables predictable resistance changes over a broad range of relative humidity levels.

Conversely, the response of the sensor (Figure 6E) increases linearly with RH, reflecting a consistent and proportional change in resistance with rising humidity. The linear fit for resistance and response are described by the equations *y* = 2.35 − 0.12*x* and *y* = 0.085*x* + 1.06, respectively, where *x* represents RH. The high correlation coefficient (*R*^2^ = 0.98) indicates the strong linearity of these relationships, affirming the sensor’s reliability in providing accurate humidity measurements. Such strong linear correlations are advantageous, as they facilitate straightforward calibration and interpretation, making the sensor suitable for applications requiring precise, continuous monitoring of humidity levels. Understanding these response characteristics is crucial for optimizing sensor performance and reliability across diverse conditions.

#### 3.2.4. Reproducibility, Reversibility, and Selectivity

To assess the reproducibility and reversibility of the chitosan-based humidity sensor, we conducted repeated resistance measurements across different RH levels, as illustrated in Figure 6F. The sensor was exposed to a sequence of response and recovery cycles over a range from 6% to 97% RH, with each RH level tested in triplicate. The sensor demonstrated a consistent resistance profile across all cycles, characterized by minimal deviation between individual curves. This high level of consistency highlights the sensor’s excellent reproducibility, confirming that it can reliably detect humidity changes across multiple cycles without significant signal drift or degradation. The minimal variation in the response curves further demonstrates the sensor’s excellent reversibility, as it can reliably return to baseline resistance levels after each humidity exposure cycle and desorption cycle. This reversibility is critical for maintaining accuracy and reliability in dynamic environments where humidity conditions fluctuate frequently. The sensor’s performance indicates that its structural and functional integrity remains uncompromised even after repeated exposure to varying humidity levels, a key feature for long-term usability.

#### 3.2.5. Statistical and Selectivity Analysis

**Figure 7:**
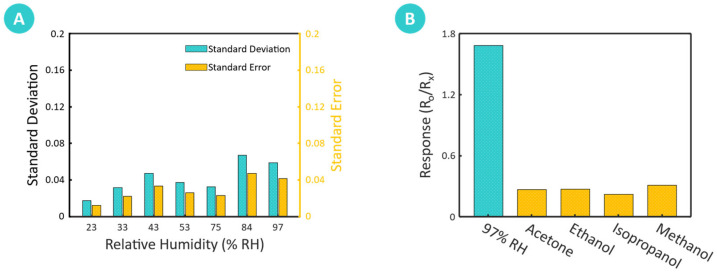
Statistical analysis, selectivity, and IoT demonstration of the biomass-derived chitosan-based humidity sensor. (**A**) Statistical analysis displaying standard deviation (SD) and standard error of the mean (SEM) for resistance measurements across varying relative humidity levels (6% to 97% RH). The low SD and SEM values indicate consistent performance with minimal variability, underscoring the reliability and stability of the developed sensor. (**B**) Response of the chitosan-based humidity sensor to various volatile organic compounds (VOCs)—acetone, ethanol, isopropanol, and methanol—highlighting the sensor’s high selectivity towards water vapor with minimal interference from VOCs.

The statistical analysis presented in Figure 7A provides important information regarding the reliability and consistency of the developed chitosan-based humidity sensor when exposed to varying relative humidity (RH) levels ranging from 6% to 97%. The calculated standard deviations (SD) of 0.018, 0.032, 0.048, 0.037, 0.033, 0.067, and 0.059, as well as the standard errors of the mean (SEM) of 0.013, 0.023, 0.034, 0.027, 0.023, 0.048, and 0.042, respectively, indicate low variability in resistance measurements for each RH level. These low standard deviations reflect the precision and reproducibility of the sensor’s performance across varying humidity levels, which are essential for consistent operation in real-world applications.

Such consistency suggests that the sensor can reliably produce stable measurements, supporting its suitability for applications that require high measurement accuracy, including environmental monitoring and industrial humidity control. Furthermore, low measurement variability minimizes uncertainty, increasing confidence in the sensor’s ability to deliver accurate and reliable humidity readings under different environmental conditions. The robustness indicated by these low SD values implies that the sensor experiences minimal performance drift or fluctuation, reinforcing its stability and making it dependable for long-term use. Figure 7B highlights the selectivity of the chitosan-based humidity sensor by displaying its responses to different volatile organic compounds (VOCs), specifically acetone, ethanol, isopropanol, and methanol, in comparison to water vapor. The bar chart reveals that the sensor demonstrates a markedly higher sensitivity to water vapor than to these interference gases. This selective response is beneficial for humidity-sensing applications, as it suggests that the sensor can effectively distinguish water vapor from other VOCs, even in environments with potential gaseous contaminants. This distinct response profile reflects the sensor’s inherent material properties, such as the strong hydrogen bonding affinity of chitosan’s hydroxyl and amine functional groups, which favor interactions with water vapor over other VOCs.

The sensor’s high selectivity is a critical advantage for humidity-sensing applications, as it ensures accurate detection of water vapor without interference from other atmospheric substances. This characteristic is particularly valuable in indoor air quality monitoring, where the presence of VOCs from household chemicals or industrial emissions could otherwise skew humidity readings. Similarly, the sensor’s ability to resist cross-sensitivity enhances its reliability in agricultural storage applications, where precise moisture control is vital for preventing spoilage and maintaining product quality. Moreover, this selective response expands the practical utility of the sensor across diverse fields, including environmental monitoring, healthcare, and industrial process regulation, where distinguishing water vapor from other gaseous components is critical. By minimizing false positives and ensuring accurate measurements, the chitosan-based humidity sensor sets itself apart as a robust and reliable tool for advanced sensing systems. While the chitosan-based sensor exhibited high selectivity for water vapor over VOCs, its sensitivity to other environmental contaminants, such as acidic or basic vapors, warrants further investigation. These contaminants could potentially interfere with the sensing mechanism and impact the sensor’s accuracy. Additionally, although the fabrication method used in this study (drop casting) is straightforward and effective for laboratory-scale production, scaling up the technology for industrial applications may require alternative techniques like spin coating or roll-to-roll processing. These methods would enhance the uniformity and scalability of the production process, making it feasible for large-scale manufacturing.

### 3.3. Sensing Mechanism of the Biomass-Derived Chitosan-Based Humidity Sensor

The humidity-sensing mechanism of chitosan is rooted in its molecular interactions with atmospheric water molecules, primarily through hydrogen bonding and proton conduction processes. The protonated amino and hydroxyl groups present in the chitosan structure enable it to adsorb and desorb water molecules depending on the relative humidity (RH) of the environment. These properties, combined with the material’s ability to conduct protons under different humidity conditions, facilitate its use as a sensitive and reversible humidity sensor. The humidity-sensing capability is largely driven by the protonated amino groups in chitosan, which are formed when the material is dissolved in an acidic medium such as acetic acid.

The reaction, as shown in Equation (3) [44], involves the protonation of amino groups:

R–NH_2_ + CH_3_COOH → R–NH_3_^+^ + CH_3_COO^−^ (3)

**Figure 8:**
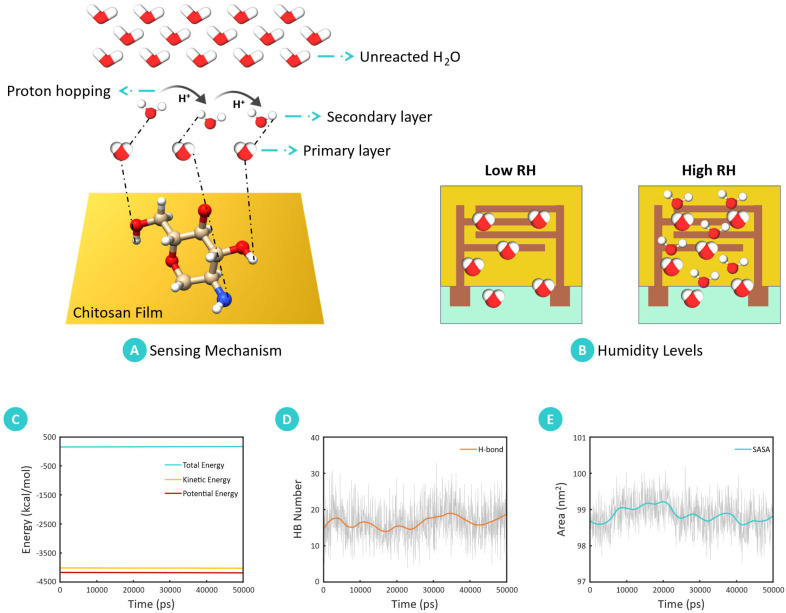
Humidity-sensing mechanism and molecular interaction in the chitosan-based humidity sensor. (**A**) Schematic representation of the humidity-sensing mechanism, detailing the interaction between chitosan and water molecules. The illustration highlights the primary and secondary layers of water molecule adsorption, the formation of hydrogen bonds, proton hopping, and unreacted molecules of water. (**B**) The impact of humidity levels on the developed chitosan-based humidity sensor. In high-humidity conditions, the sensing material interacts with a significant number of water molecules, whereas in low-humidity conditions, the interaction is minimal. (**C**) Presents a plot of the energy evolution during the simulated chitosan solvation process, demonstrating successful equilibration and a constant energy state throughout the simulated timesteps. (**D**) The plot of the quantity of hydrogen bonds formed between the structure of chitosan and the water molecules over the simulated timesteps, with an average of 17 hydrogen bonds observed. (**E**) The solvent-accessible surface area, with an indicated average of 99 nm^2^.

These protonated amino groups readily form hydrogen bonds with water molecules, while the hydroxyl groups along the chitosan backbone contribute additional hydrogen bonds with atmospheric water. This dual bonding capability enables the reversible adsorption (humidification) and desorption of water molecules (dehumidification) on the chitosan film’s surface, enabling the sensor to adapt to RH variations effectively [2]. As illustrated in Figure 8, the molecular interaction between chitosan and adsorbed molecules of water directly influences the film’s conductivity. Thus, two primary proton conduction mechanisms underlie the conductivity changes observed in chitosan films—the Grotthuss mechanism and the packed-acid mechanism [45]. Under high humidity conditions, the Grotthuss mechanism dominates. Protons (H^+^ ions) “hop” along a hydrogen bond network formed between molecules of water and protonated amino groups on the chitosan chain (Figure 8A). This proton hopping significantly increases conductivity in hydrated chitosan films, allowing for quick adaptation to RH changes. Additionally, the limited swelling of chitosan under high humidity contributes to reduced hysteresis, maintaining stability in dynamic humidity environments [2].

In contrast, at low humidity levels, the packed-acid mechanism explains proton conductivity. Under these conditions, protons migrate via acid–acid interactions without involving water molecules, resulting in a network of weak hydrogen bonds among proton donors in the chitosan matrix, as illustrated in Figure 8B. This alternative conduction pathway enables the sensor to maintain some degree of proton conductivity even in dry conditions, allowing it to respond to minimal humidity changes.

The molecular dynamics (MD) simulation further elucidated the chitosan humidity-sensing mechanism by examining interactions at the molecular level. Figure 8C illustrates the energy evolution during the simulation, conducted under the NPT ensemble. The stability of the system was confirmed by the consistent values of potential, kinetic, and total energy over the simulation timeline. Additionally, a minor reduction in simulation cell volume (0.21 Å) indicated further stabilization, supporting the robustness of the simulation model. Detailed analysis of the equilibrated chitosan structure revealed the formation of hydrogen bonds between the chitosan chains and surrounding water molecules, which corroborates the experimentally observed sensing mechanism. As shown in Figure 8D, the simulation calculated an average of 17 hydrogen bonds between chitosan and water molecules, highlighting the chitosan’s affinity for water through hydrogen bonding. The formation of hydrogen bonds between chitosan and water molecules, observed both experimentally and in MD simulations, aligns with the sensing mechanism wherein chitosan’s hydrophilic properties enable effective interaction with ambient humidity.

The solvent-accessible surface area (SASA) of the chitosan chain, computed as 99 nm^2^, further substantiates its high sensitivity to relative humidity (RH). A larger SASA enhances the number of available interaction sites for water molecules, increasing the material’s responsiveness to humidity variations. Figure 8E presents the SASA over the simulation period, emphasizing the chitosan’s potential for water molecule contact, crucial for rapid and reversible humidity sensing. The results from MD simulations corroborate the experimental findings, demonstrating that the structure of chitosan, its ability to form hydrogen bonds, and proton mobility mechanisms underpin its humidity-sensing capabilities. This dual mechanism—wherein the Grotthuss mechanism prevails at high humidity and the packed-acid mechanism at low humidity—facilitates reliable and responsive humidity detection, making chitosan an effective sensing material for humidity-sensing applications.

In the fabricated chitosan-based humidity sensor, the thin layer of polyvinyl alcohol (PVA) serves as a crucial substrate within the laminate composite, contributing significantly to the overall sensing mechanism. The inherent hygroscopic nature of PVA enhances the sensor’s ability to interact with atmospheric water molecules. By absorbing moisture from the surrounding environment, the PVA layer facilitates the adsorption process, creating an interface that promotes efficient water molecule interaction with the chitosan film. This moisture retention capability of PVA aids in maintaining a stable and uniform distribution of water molecules across the sensor surface, optimizing the conductivity changes induced by the chitosan layer. Thus, the hygroscopic properties of the PVA substrate play a crucial role in the sensor’s performance by ensuring uniform moisture absorption, which aids in minimizing hysteresis. This characteristic contributes to the sensor’s ability to deliver consistent and accurate readings during cyclic humidity transitions. However, prolonged exposure to elevated humidity levels may compromise the structural stability of the PVA layer, potentially affecting the sensor’s durability. Additionally, flexibility and compatibility of PVA with the laminate structure ensure mechanical stability and durability, enabling the sensor to maintain consistent performance under varying humidity conditions. Thus, the inclusion of a hygroscopic PVA substrate not only supports the structural integrity of the sensor but also amplifies its sensitivity to humidity changes, making this substrate integral to the design and functionality of the developed sensor.

The moisture content of the chitosan-based humidity sensor was determined in order to quantify its water absorption capacity, which plays a significant role in its sensing mechanism. Using an analytical balance (model ADB200-4-EA, KERN & SOHN GmbH, Balingen, Germany) with an accuracy of 0.1 mg, the initial weight of the sensor was measured. The sensor was subsequently dried at 60 °C for 6 h to obtain its dry mass. This process was repeated three times, and the average values were recorded. The moisture content was calculated as the percentage difference between the initial and dry weights relative to the initial weight and was found to be 3.24 ± 0.59%. While the hygroscopic nature of the developed sensor enhances the developed sensor’s sensitivity to atmospheric moisture, prolonged exposure to high humidity could result in gradual degradation, affecting the sensor’s structural integrity and long-term stability. Future studies will focus on addressing this limitation by evaluating the sensor’s performance under extended high-humidity conditions. The use of alternative or modified substrates with enhanced moisture resistance can potentially mitigate any adverse effects on performance.

### 3.4. Comparative Analysis of the Biomass-Derived Chitosan-Based Humidity Sensor

To evaluate the performance of the biomass-derived chitosan-based humidity sensor developed in this study, a comparative analysis with existing polymer-based resistive-type humidity sensors was conducted, as summarized in Table 1. This comparison encompasses key parameters, including the sensing material choice, the fabrication method used, the relative humidity measurement range, the response and recovery times, and relevant references, providing a comprehensive perspective on how the developed sensor performs relative to other sensor materials and designs.

Existing polymer-based humidity sensors typically use pristine polymers such as polypyrrole (Ppy), cellulose paper (CP), polyvinyl alcohol (PVA), chitosan (CS), and copolymer composites such as methyl methacrylate (MMA) with [3-(methacrylamino)propyl] trimethyl ammonium chloride (MAPTAC) and cellulose paper (CP) modified with glycidyl trimethyl ammonium chloride (EPTAC). These materials have been widely explored due to their diverse chemical and physical properties, which influence their sensing capabilities. The fabrication methods for these sensors vary widely and include techniques such as oxidative polymerization, facile pasting, spin coating, drop casting, wet coupling, and copolymerization. Among these, drop casting, as employed in the current study, stands out for its simplicity, cost-effectiveness, and scalability.

The measurement range is a critical performance indicator for humidity sensors, as it determines their applicability across diverse environmental conditions. The humidity sensor developed in this study demonstrates an extensive measurement range from 6% to 97% RH, significantly surpassing many existing polymer-based sensors. For example, Ppy-based sensors [46] measure RH in a narrower range of 30–90%, while cellulose–polyaniline (Cell–PANI) sensors [48] are limited to 30–50% RH. Similarly, the MMA–MAPTAC copolymer [49] and CP [16] sensors also exhibit restricted ranges compared to the developed humidity sensor, with ranges of 10–90% and 41.1–91.5% RH, respectively. Only certain sensors, such as the PVA [47], CS [2], and EPTAC–CP [50], approach or match the wide range of the developed humidity sensor, which indicates its versatility in diverse humidity conditions. Response and recovery times are critical indicators of a humidity sensor’s dynamic performance, reflecting its ability to adapt to rapid environmental changes. The biomass-derived chitosan-based humidity sensor developed in this study shows impressive dynamic performance, with a fast response time of 18.22 s and a corresponding recovery time of 22.39 s.

This compares favorably with other polymer-based sensors, particularly those with slower response times. For instance, Ppy-based sensors require 128 s for both response and recovery [46]. Similarly, PVA-based sensors exhibit a response time of 224.6 s and a recovery time of 56.3 s [47]. The CS-based sensor also demonstrates a fast response time of 21 s with a recovery time of 25 s, similar to the developed chitosan-based humidity sensor [2]. However, other sensors like MMA–MAPTAC [49] and EPTAC–CP [50] exhibit comparatively slower recovery times, at 150 and 188 s, respectively. The rapid response and recovery in the developed sensor indicates a high sensitivity to humidity changes, which is advantageous for real-time monitoring.

The biomass origin of the chitosan used in the developed humidity sensor offers additional environmental benefits, aligning with current trends in sustainable sensor development. Unlike other humidity sensors utilizing pristine polymers, the sensor leverages renewable chitosan extracted from biowaste, which not only reduces reliance on synthetic materials but also enhances the sensor’s appeal for applications where environmental impact is a key consideration. This aligns with broader research efforts focused on creating eco-friendly, high-performance humidity sensors using sustainable materials. By transforming agricultural and industrial byproducts into high-value sensing materials, the biomass-derived chitosan-based humidity sensor embodies the principles of circular economy and resource efficiency. This innovative use of biowaste aligns with broader research trends focused on eco-friendly sensor development, paving the way for greener alternatives to traditional synthetic materials. The extraction of chitosan from mealworm biomass offers a dual benefit: it mitigates environmental pollution associated with biowaste while providing a cost-effective, biodegradable, and biocompatible material for advanced sensing applications. Furthermore, the sensor’s reliance on naturally sourced materials enhances its appeal in applications where environmental impact is a key consideration, such as environmental monitoring, smart agriculture, and healthcare. Its sustainable fabrication method and high performance make it a compelling example of how green materials can meet the technical demands of modern sensor technologies. The developed humidity sensor not only exemplifies the integration of sustainability and innovation but also sets a benchmark for future research focused on environmentally conscious sensor systems.

### 3.5. Integration of the Biomass-Derived Chitosan-Based Humidity Sensor in an IoT Framework

**Figure 9:**
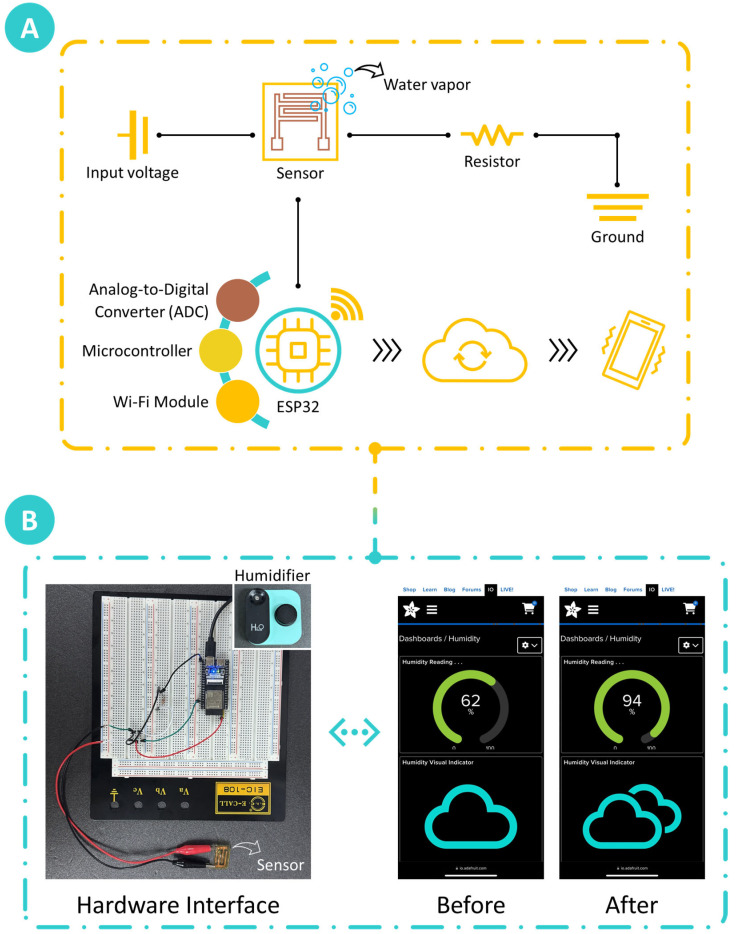
IoT-enabled system setup and real-time monitoring interface for the chitosan-based humidity sensor. (**A**) Schematic of the IoT configuration including the input voltage, resistor, ADC, microcontroller, and Wi-Fi module, enabling the sensor’s integration within an IoT framework. (**B**) Digital photograph of the hardware interface including the chitosan-based humidity sensor, ESP32 microcontroller, and humidifier (left) (model G15 USB Humidifier, Dongguan Leang Electronics Co., Ltd., Dongguan, China). Remote monitoring dashboard on Adafruit IO, displaying RH readings as a percentage gauge and visual indicator, showcasing the sensor’s responsiveness to environmental humidity levels and suitability for real-time IoT applications (right).

To validate the feasibility and performance of the chitosan-based humidity sensor within an Internet of Things (IoT) framework, a comprehensive system was developed to enable real-time monitoring and wireless transmission of humidity data. The setup, illustrated in Figure 9A, integrates a series of interconnected electronic components that convert the sensor’s analog output into digital signals, enabling wireless data transmission and remote display on a mobile device. The demonstration began with the provision of a stable 3.3 V power supply to the humidity sensor, ensuring consistent operation throughout the testing period. The sensor was connected in series with a resistor to monitor current variations induced by changes in the sensor’s resistance, which varied in response to fluctuations in water vapor levels. These resistance changes produced corresponding voltage variations across the resistor, creating a measurable output signal that directly reflected the relative humidity (RH) in the environment.

This analog voltage output was subsequently fed into an analog-to-digital converter (ADC), which digitized the sensor’s real-time voltage variations. The ADC played a crucial role in translating the analog outputs into digital formats to be further processed. Once digitized, the output data were directed to an ESP32 microcontroller (model ESP32-S3, Shenzhen Freenove Creative Technology Co., Ltd., Shenzhen, China), which acted as the central processor of the IoT setup. The microcontroller interpreted the voltage signal and converted it into RH values, which were then prepared for wireless transmission. To enable real-time monitoring, the data were wirelessly transmitted via a Wi-Fi module embedded in the system. This module enabled seamless transmission of RH readings to a remote monitoring device (iPhone 15 Pro Max), allowing for continuous monitoring without the constraints of a wired connection. As shown in Figure 9B, this setup illustrates the effective integration of the chitosan-based humidity sensor into a functional IoT system, demonstrating its capability for continuous, real-time environmental monitoring.

In the demonstration, a commercially available humidifier was used to elevate the ambient RH level from an initial reading of 62% RH (baseline room humidity) to a higher level (94% RH), effectively showcasing the sensor’s responsiveness to water vapor changes. The acquired RH data were integrated with an online dashboard built using the Adafruit IO platform [51], which offers a versatile solution for IoT device management and real-time data visualization. The dashboard displayed the RH readings on a user-friendly interface featuring a percentage gauge for humidity reading and a visual indicator, providing a clear representation of the sensor’s real-time response to environmental humidity changes. This capability to wirelessly display real-time measurements emphasizes the practical potential of the chitosan-based humidity sensor for continuous, remote monitoring in diverse applications. The sensor system, comprising the chitosan-based sensor, microcontroller, and Wi-Fi module, was powered by a 3.3 V supply. This low energy requirement makes it suitable for portable or battery-operated applications. IoT integration enhances the sensor’s utility by enabling real-time data acquisition, wireless transmission, and remote visualization through platforms such as Adafruit IO. These features allow for continuous monitoring without physical connections, making the system practical for diverse applications such as environmental monitoring, agriculture, and industrial automation. However, challenges such as maintaining stable connectivity in remote locations and ensuring data security in IoT networks will need to be addressed to facilitate broader adoption.

While chitosan has been widely studied as a humidity-sensing material in prior studies, this work introduces a novel approach by utilizing chitosan extracted from mealworm biomass, a renewable biowaste source. This sustainable material source represents a significant shift in sensor development, addressing the global demand for eco-friendly technologies while reducing reliance on conventional pristine polymers. Unlike conventional studies, this work integrates the chitosan-based humidity sensor into an IoT framework, allowing real-time, wireless monitoring and data visualization. The IoT integration elevates the sensor’s applicability by facilitating continuous, remote humidity monitoring in diverse fields, including smart agriculture, environmental protection, and industrial automation. This demonstrates how biowaste-derived materials can seamlessly align with cutting-edge IoT applications, paving the way for scalable, intelligent sensing systems. To further advance the understanding of chitosan as a sensing material, molecular dynamics simulations were employed to elucidate the fundamental humidity-sensing mechanism at the molecular level. The simulations provided in-depth insights into the roles of proton mobility, hydrogen bonding, and solvent-accessible surface area in modulating the sensor’s response to varying humidity levels. These findings represent a significant contribution to the scientific understanding of chitosan’s behavior in sensing applications, which has not been comprehensively addressed in previous studies. Comprehensive performance evaluations of the developed sensor revealed exceptional characteristics, including rapid response and recovery times, low hysteresis, high sensitivity, and a broad humidity measurement range (6–97% RH). Comparative analyses with other polymer-based humidity sensors highlighted the superior performance of the chitosan-based humidity sensor, particularly in terms of sustainability and practical implementation. This study bridges the gap between sustainable materials and advanced IoT technologies, offering a holistic approach to developing high-performance, environmentally friendly humidity sensors. By utilizing renewable chitosan and modern IoT frameworks, this work provides a blueprint for future developments in sustainable and intelligent sensing technologies.

## 4. Conclusions and Future Directions

In conclusion, the biomass-derived chitosan-based humidity sensor developed in this study demonstrates substantial promise as a flexible, high-performance, and environmentally sustainable solution for humidity sensing. The chitosan-based humidity sensor, fabricated using chitosan extracted from mealworm biomass, showcased high sensitivity in a broad humidity-sensing range (6–97% RH), coupled with rapid response (18.22 s) and recovery (22.39 s) times. These favorable characteristics are driven by chitosan’s inherent hydrophilicity and proton conductivity, enabling efficient and reversible adsorption (humidification) and desorption of water molecules (dehumidification). Comparative analysis with conventional polymer-based sensors illustrates the chitosan-based humidity sensor’s competitive performance, with the added benefit of sustainability due to its renewable chitosan source. The molecular dynamics simulations provided valuable insights into the underlying sensing mechanism, showing the roles of the Grotthuss and packed-acid mechanisms in modulating conductivity under varying humidity levels. Furthermore, the successful integration of the chitosan-based humidity sensor into an IoT system demonstrates its practicality for real-time, wireless environmental monitoring, making it suitable for applications such as smart agriculture, industrial automation, and climate control systems.

Future work will aim to further enhance the chitosan-based humidity sensor by exploring alternative eco-friendly composites and optimizing its structure for even faster response times and higher sensitivity. The drop-casting method employed in this study was chosen for its simplicity and suitability for laboratory-scale fabrication. However, for industrial applications, alternative methods such as spin coating, spray coating, or roll-to-roll processing could be explored. These techniques offer advantages in terms of scalability, speed, and uniformity, making them well-suited for large-scale production of chitosan-based humidity sensors. Future work will focus on optimizing the fabrication process to enable efficient and consistent industrial-scale manufacturing. Additionally, testing the sensor’s durability under prolonged exposure to extreme environmental conditions will be essential for expanding its application range. This study evaluated the sensor under controlled laboratory conditions to establish its baseline performance. However, testing its robustness under extreme environmental conditions, such as high temperatures and pollutant exposure, remains a critical area for future investigation. Assessing the sensor’s thermal stability, long-term durability, and resistance to contaminants will provide valuable insights into its suitability for harsh or dynamic environments. These aspects will be explored in subsequent studies to further validate the sensor’s practical applicability. We also aim to build upon the present study by advancing the sensing material and device design to develop a fully portable, wireless humidity-monitoring system suitable for diverse applications. Our primary focus will be on refining the instrumentation for enhanced precision and integrating machine learning (ML) algorithms to improve sensor calibration, signal processing, and data interpretation.

The use of biowaste-derived chitosan in the development of the humidity sensor aligns with the principles of sustainability by addressing two critical environmental concerns. First, it reduces dependence on synthetic polymers, which are typically produced from non-renewable petroleum-based resources. Second, it provides a valuable use for mealworm biomass, a byproduct of large-scale mealworm farming, thereby mitigating waste disposal challenges and reducing environmental pollution. This approach not only supports the circular economy but also enhances the environmental appeal of the sensor, making it a viable alternative to conventional polymer-based technologies. By leveraging biomass-derived chitosan and advancing IoT capabilities, this study underscores the potential for sustainable and scalable humidity sensors that address both environmental and technological needs in the evolving field of smart sensing. This work contributes to the evolution of smart sensing solutions that align with global trends toward sustainability and technological innovation.

## Figures and Tables

**Table 1:** Comparative analysis of the chitosan-based humidity sensor with existing polymer-based resistive-type humidity sensors.

Sensing Material	Material Source	Fabrication Method	Measuring Range (% RH)	Response/Recovery Time (s)	Reference
^a^ Ppy	Pristine	Oxidative polymerization	30–90	128/128	[46]
^b^ CP	Pristine	Facile pasting	41.1–91.5	472/19	[16]
^c^ PVA	Pristine	Spin coating	7–92	224.6/56.3	[47]
^d^ CS	Pristine	Drop casting	11–95	21/25	[2]
^e^ Cell–PANI	Pristine	Wet coupling	30–50	90/87	[48]
^f^ MMA–MAPTAC	Pristine	Copolymerization	10–90	45/150	[49]
^g^ EPTAC–CP	Pristine	Facile solution	11–95	25/188	[50]
^h^ CHS	Biomass	Drop casting	6–97	18.22/22.39	Present study

^a^ Polypyrrole composite (Ppy), ^b^ cellulose paper (CP), ^c^ poly(vinyl alcohol) (PVA), ^d^ chitosan (CS), ^e^ cellulose–polyaniline (Cell–PANI), ^f^ methyl methacrylate (MMA)–[3-(methacrylamino)propyl] trimethyl ammonium chloride (MAPTAC) copolymer, ^g^ glycidyl trimethyl ammonium chloride (EPTAC)-modified cellulose paper (CP), ^h^ biomass-derived chitosan-based humidity sensor (CHS).

## Data Availability

The data presented in this study are available from the corresponding authors upon request.
